# A Comprehensive Review of Nanotechnological Innovations in Cancer: from Molecular Pathways to Clinical Applications

**DOI:** 10.7150/jca.126813

**Published:** 2026-02-04

**Authors:** Saqib Nawaz, Wei Ho, Asif Nawaz, Sajid Ur Rahman, Ayesha Zahid, Khalid J. Alzahrani, Ibrahim F. Halawani, Fuad M. Alzahrani, Chien-Chin Chen, Abdul Qadeer

**Affiliations:** 1 Anhui Province Key Laboratory of Veterinary Pathobiology and Disease Control, College of Veterinary Medicine, Anhui Agricultural University, Hefei 230036, PR China.; 2 Department of Orthopaedics, Ditmanson Medical Foundation Chia-Yi Christian Hospital, Chiayi 600, Taiwan.; 3 Faculty of Agriculture, The University of Agriculture, Dera Ismail Khan, Dera Ismail Khan 29111, Pakistan.; 4 Department of Public Health and Emergency Management, School of Medicine, Southern University of Science and Technology, Shenzhen, China.; 5 Institute for Biomedicine and Glycomics, Griffith University, Gold Coast, Queensland, Australia.; 6 School of Pharmacy and Medical Sciences, Griffith University, Gold Coast, Queensland, Australia.; 7 Department of Clinical Laboratories Sciences, College of Applied Medical Sciences, Taif University, P.O. Box 11099, Taif 21944, Saudi Arabia.; 8 Department of Pathology, Ditmanson Medical Foundation Chia-Yi Christian Hospital, Chiayi 600, Taiwan.; 9 Department of Cosmetic Science, Chia Nan University of Pharmacy and Science, Tainan 717, Taiwan.; 10 Doctoral Program in Translational Medicine, National Chung Hsing University, Taichung 402, Taiwan.; 11 Department of Biotechnology and Bioindustry Sciences, College of Bioscience and Biotechnology, National Cheng Kung University, Tainan, 701, Taiwan.; 12 Department of Cell Biology, School of Life Sciences, Central South University, Changsha, China.; 13 School of Medicine, Shandong Xiehe University, Jinan, Shandong, 250109, China.

**Keywords:** nanoparticle, PI3K/Akt/mTOR, Wnt/β-catenin, MAPK/ERK, p53, cancer therapy

## Abstract

This review offers an inclusive overview of current cancer treatment options. It examines emerging trends in molecular mechanisms, therapeutic challenges, and nanotechnological innovations that may significantly improve patient outcomes. Cancer is a complex disease characterized by unregulated cellular proliferation, invasion of adjacent tissues, and metastasis. It remains a leading cause of death worldwide, with increasing incidence attributed to factors such as an aging demographic, lifestyle habits, and environmental stimuli. This review examines various cancer types, their associated risk factors, and critical molecular pathways, with the PI3K/Akt/mTOR and Wnt/β-catenin pathways, that facilitate cancer progression. The intricate process of metastasis, the leading cause of cancer fatalities, is examined in depth. The discussion encompasses a comprehensive analysis of diagnostic methods and conventional treatment modalities, including surgical intervention, radiation therapy, chemotherapy, and targeted therapies, and addresses their limitations, including toxicity, drug resistance, and unintended side effects. The advent of nanoparticle-based platforms in cancer therapy has facilitated significant progress in targeted drug delivery, enhanced imaging for prompt diagnosis, and the advancement of treatments, such as photothermal and photodynamic therapies. Nanoparticles may provide a vital function in overcoming drug resistance, thereby improving treatment effectiveness. Furthermore, this review emphasizes cancer prevention strategies, which include lifestyle changes and vaccination efforts. The oncological treatment landscape is poised for transformation through advancements in precision medicine, gene therapy, and artificial intelligence. Incorporating nanotechnology into these emerging strategies holds substantial potential for developing more personalized and effective cancer therapies with reduced adverse effects.

## 1. Introduction

Cancer is a complex group of diseases characterized by uncontrolled cellular proliferation, invasion of adjacent tissues, and metastatic spread to distant organs. It is a leading cause of global mortality, with a multifaceted etiology involving genetic, epigenetic, and environmental factors and more than 100 recognized types 1. Population growth and aging demographics contribute to rising cancer incidence. Additionally, modifiable lifestyle factors, including smoking, poor dietary patterns, and physical inactivity, further elevate cancer risk 2. At the cellular level, it arises from genetic mutations and epigenetic changes that disrupt normal regulatory mechanisms, leading to unregulated cell proliferation, resistance to programmed cell death (apoptosis) 6, and the ability to invade adjacent tissues 6. Tumor cells frequently develop additional characteristics, including resistance to anti-proliferative signals, the ability to sustain angiogenesis, and evasion of immune detection 3.

### 1.1 Epidemiology

**Figure 1:**
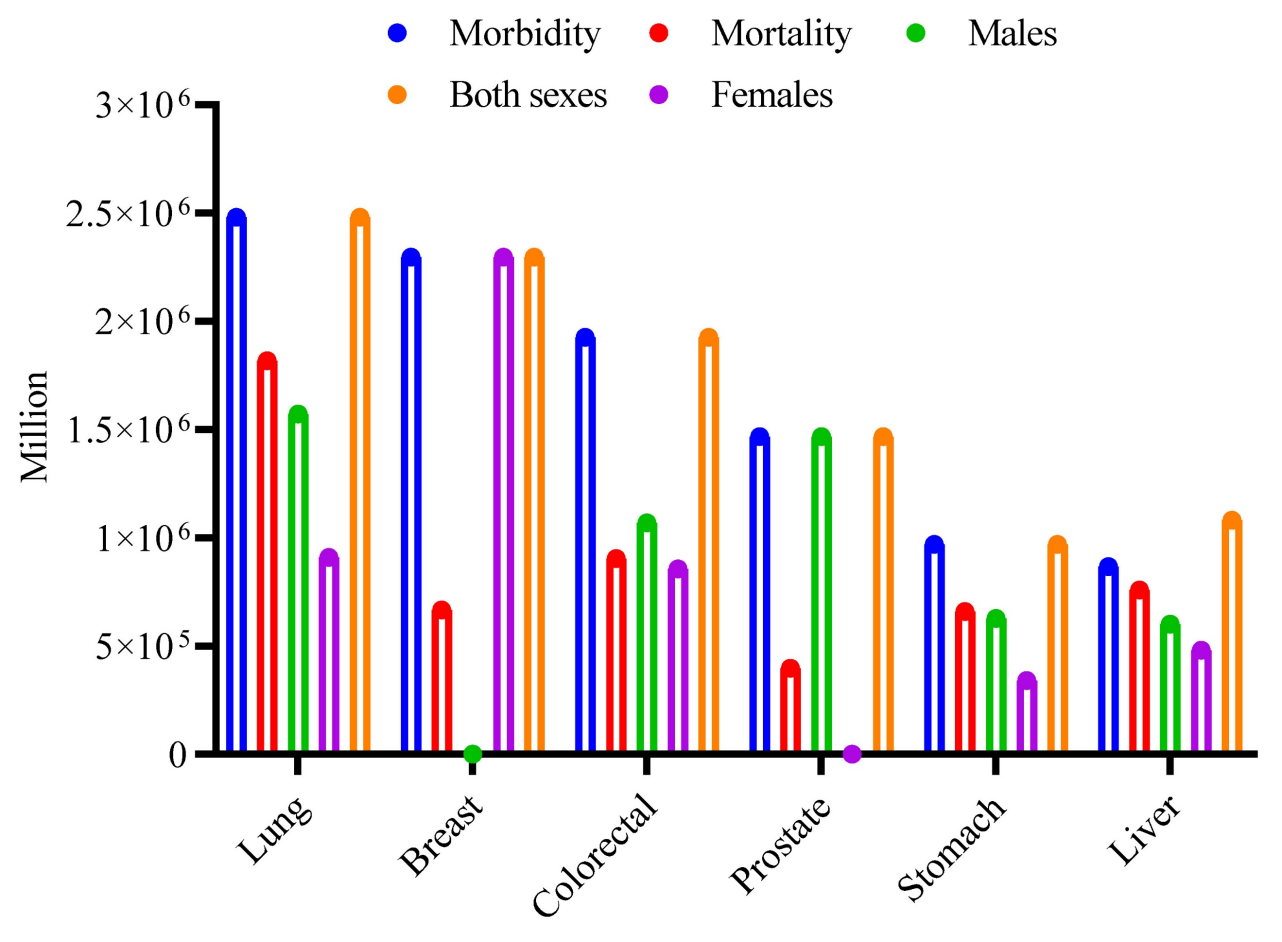
Global burden of major cancer types in 2022. The clustered bar chart shows the estimated number of new cases (Incidence) and deaths (Mortality) worldwide for the six most common cancers, as reported by GLOBOCAN 2022. The first two bars for each cancer type represent the total for both sexes. The subsequent three bars show the sex-specific incidence: cases in males only, cases in females only, and the combined total for both sexes. Data values are in millions 5.

Cancer is a leading cause of global mortality, accounting for about 9.7 million deaths in 2022 4, 5. In the year 2024, about 2,001,140 new cancer cases were identified in the United States, with an anticipated 611,720 deaths due to the illness. The most common forms of cancer include bladder, breast, colorectal, endometrial, kidney and renal pelvis, leukemia, liver, lung and bronchus, melanoma, non-Hodgkin lymphoma, pancreatic, prostate, and thyroid. Prostate, lung, and colorectal cancers in males are predicted to account for around 48% of all cancer cases identified in 2024 6. For women, the leading cancers are breast, lung, and colorectal, which together are anticipated to constitute approximately 51% of all new cancer diagnoses in that year, as reported by the WHO, 2024 7. Global cancer incidence and mortality for the most common types in 2022 are summarized in (Fig. 1), based on GLOBOCAN data. For both sexes combined, lung cancer had the highest incidence (2.48 million new cases), followed by breast (2.30 million), colorectal (1.93 million), prostate (1.47 million), stomach (0.97 million), and liver (0.87 million) cancers. Mortality was also highest for lung cancer (1.82 million deaths), followed by colorectal (0.90 million), liver (0.76 million), stomach (0.66 million), breast (0.67 million), and prostate (0.40 million) cancers. The figure further disaggregates the incidence for these six cancer types by sex, highlighting, for example, the high incidence of breast cancer in females and lung cancer in males 5.

### 1.2 Major types

Cancers are classified by cellular origin, which influences their behavior, prognosis, and treatment. The primary classifications comprise carcinomas, sarcomas, leukemias, lymphomas, and melanomas, each with unique characteristics, examples, and associated risk factors. Carcinomas represent the most common category of cancer, constituting approximately 80-90% of all cancer cases. These tumors arise from epithelial cells that line both internal and external body surfaces, including the skin, the linings of the digestive and respiratory systems, and the parenchyma of various organs 8. Given their anatomical positioning, carcinomas are often subjected to environmental carcinogens. This group is further classified according to the specific type of epithelial cell involved. Adenocarcinomas, for instance, develop in glandular tissues and are frequently observed in the breast, prostate, colon, pancreas, and lungs 9.

Squamous cell carcinomas, on the other hand, originate from squamous epithelial cells and can be found in the skin, cervix, and the linings of the head and neck region 10. Additional subtypes include transitional cell carcinoma of the bladder, basal cell carcinoma of the skin (the most prevalent yet least aggressive form of skin cancer), and renal cell carcinoma of the kidney 11. Significant risk factors for carcinomas are typically environmental or related to lifestyle choices, such as tobacco use (which is strongly associated with lung, bladder, and pancreatic cancers), exposure to ultraviolet (UV) radiation (linked to skin carcinomas), high intake of red meat (associated with colorectal cancer), and infections like the human papillomavirus (HPV), a leading cause of cervical carcinoma 12. Furthermore, genetic factors, including mutations in the Breast Cancer gene 1 (BRCA1) and Breast Cancer gene 2 (BRCA2), significantly increase the risk of breast and ovarian cancers 8, 13.

In contrast to the prevalent carcinomas, sarcomas are relatively infrequent malignancies, accounting for approximately 1% of adult cancers. They develop from mesenchymal cells, which form the body's connective and supportive tissues, including bone, blood vessels, cartilage, fat, and muscle. Sarcomas are classified according to the tissue from which they originate. For example, liposarcoma originates from adipose cells, smooth muscle (leiomyosarcoma), and skeletal muscle (rhabdomyosarcoma). Bone sarcomas encompass osteosarcoma and Ewing sarcoma. Sarcomas are less associated with lifestyle factors and more associated with genetic syndromes, including Li-Fraumeni syndrome, as well as prior exposure to radiation therapy 14-16.

Leukemias are classified as cancers that affect the blood and bone marrow. These malignancies are characterized by the uncontrolled growth of abnormal white blood cells in the bone marrow 17. This abnormal proliferation disrupts normal blood cell production, leading to complications such as infections, anemia, and bleeding disorders. Leukemias are classified based on their rate of progression, which can be either acute or chronic, as well as the specific type of blood cell, namely myeloid or lymphoid 18. Leukemia is classified into four types: acute lymphoblastic leukemia (ALL), acute myeloid leukemia (AML), chronic lymphocytic leukemia (CLL), and chronic myeloid leukemia (CML) 19. Several risk factors have been identified, including genetic mutations, high-level radiation exposure, and exposure to specific chemicals, such as benzene 20, 21.

Lymphomas are malignancies that arise from the lymphatic system, a vital component of the immune system that primarily targets lymphocytes, a kind of white blood cell 22. These tumors are primarily classified into two types: Hodgkin Lymphoma (HL) and Non-Hodgkin Lymphoma. Reed-Sternberg cells are characteristic of Hodgkin Lymphoma and its subgroups, including Classical HL 23. Non-Hodgkin Lymphoma is more common and comes in a variety of types, including Diffuse Large B-cell Lymphoma (DLBCL) and Follicular Lymphoma 24. Key risk factors for developing lymphoma include a compromised immune system, commonly caused by Human Immunodeficiency Virus/Acquired Immunodeficiency Syndrome (HIV/AIDS), infections with viruses such as Epstein-Barr virus (EBV), and the use of immunosuppressive medicines 25.

Furthermore, melanomas are particularly aggressive skin cancers that originate in melanocytes, the cells that produce melanin, the pigment that gives skin its color. Although they account for a tiny proportion of skin cancer diagnoses, they are responsible for the bulk of skin cancer deaths due to their high proclivity for metastasis if not detected and treated immediately 26. Melanoma has several subtypes, including the most common, Superficial Spreading Melanoma, the aggressive Nodular Melanoma, Lentigo Maligna Melanoma, which is typically detected in sun-damaged skin, and the uncommon Acral Lentiginous Melanoma, which develops on the palms, soles, or beneath the nails 27. Exposure to ultraviolet (UV) radiation from sunlight or tanning beds is the primary risk factor for melanoma, and individuals with pale skin, light hair, freckles, or a family history of melanoma are at a higher risk of developing it 28, 29 (Table 1).

This review examines the essential elements of cancer, from its molecular underpinnings to its manifestations in various tissues. This review commences with an overview of the principal cancer types and the risk factors associated with their development, followed by a detailed examination of the molecular pathways that drive cancer progression 32, the mechanisms underlying metastasis, and contemporary approaches to diagnosis and treatment.

### 1.3 Risk factors of cancer

A range of factors promotes cancer development, including a complex interplay of endogenous, environmental, and lifestyle factors. Host factors encompass genetic and epigenetic alterations, age, immune function, and metabolic state; each can influence susceptibility to carcinogenesis. Environmental factors encompass solar and other types of radiation, dietary components, environmental contaminants, and carcinogens. Infectious agents form a critical subset of environmental risk factors that contribute to cancer development through mechanisms including chronic inflammation, genomic damage, or oncogene insertion. Among viruses, well-known oncogenic pathogens include HBV (Hepatitis B virus) and HCV (Hepatitis C virus), which are associated with liver cancer, and HPV (human papillomavirus), linked to cervical and oropharyngeal cancers 33, 34. EBV (Epstein-Barr virus) is implicated in nasopharyngeal carcinoma and certain lymphomas, HTLV (Human T-cell lymphotropic virus type 1), KSHV (Kaposi's sarcoma-associated herpesvirus), and others 35. HIV (Human immunodeficiency virus type 1), which increases cancer risk primarily through immunosuppression, facilitates the oncogenic activity of co-infecting viruses such as KSHV and HPV 36.

**Figure 2:**
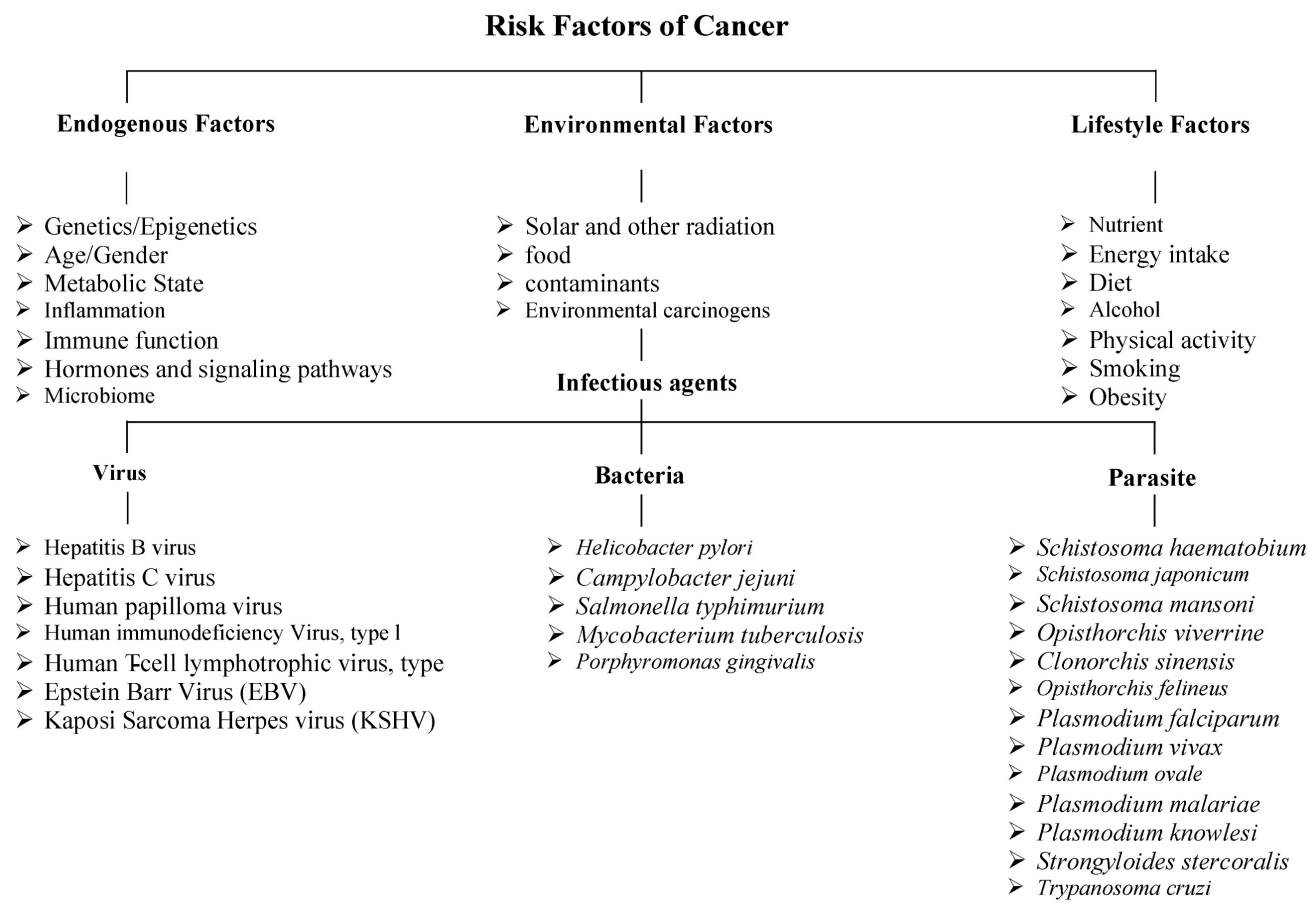
A diagram illustrating the risk factors of cancer.

Key bacterial oncogenic agents include *Helicobacter pylori*, which is strongly associated with gastric cancer, *Campylobacter jejuni*, *Salmonella typhimurium*, *Mycobacterium tuberculosis*, and *Porphyromonas gingivalis.* Parasitic infections contribute significantly to endemic regions; *Schistosoma haematobium* is linked to bladder cancer, while liver flukes such as *Clonorchis sinensis*, *Schistosoma japonicum*, *Schistosoma mansoni*, *Opisthorchis viverrini*, and *Opisthorchis felineus* are associated with cholangiocarcinoma. Other parasites include *Plasmodium* spp., *Strongyloides stercoralis*, and *Trypanosoma cruzi* 37. Exposure to carcinogenic chemical substances further elevates cancer risk. Tobacco smoke contains polycyclic aromatic hydrocarbons (PAHs) implicated in lung, bladder, and pancreatic cancers. Alcohol consumption acts synergistically with tobacco, contributing to liver, breast, and head and neck cancers 38. Aflatoxins produced by *Aspergillus* species are potent hepatocarcinogens. Industrial and occupational exposures to chemicals like benzene, asbestos, and formaldehyde are linked to leukemia, mesothelioma, and nasopharyngeal cancer. Physical environmental exposures, such as ultraviolet (UV) radiation from sunlight, contribute to the development of melanoma and other skin cancers. In contrast, ionizing radiation (e.g., X-rays, radon) is associated with an increased risk of thyroid cancer and leukemia 39. Occupational exposures in the medical and nuclear fields increase lifetime cancer risk. Lifestyle factors, including nutrient intake, energy consumption, diet, alcohol use, physical activity levels, smoking, and obesity, modulate cancer susceptibility. Obesity exerts carcinogenic effects through chronic inflammation and altered hormone levels, and is associated with at least 13 cancer types 40. Sedentary behavior, poor sleep hygiene, and chronic psychological stress also impact tumor development. Age remains the most significant risk factor, as cumulative genetic mutations and cellular damage increase over time. This integrated framework highlights the multifactorial nature of cancer risk, spanning intrinsic genetic makeup to external environmental exposures and modifiable lifestyle factors (Fig. 2) 41.

### 1.4 Pathogenesis of cancer

**Figure 3:**
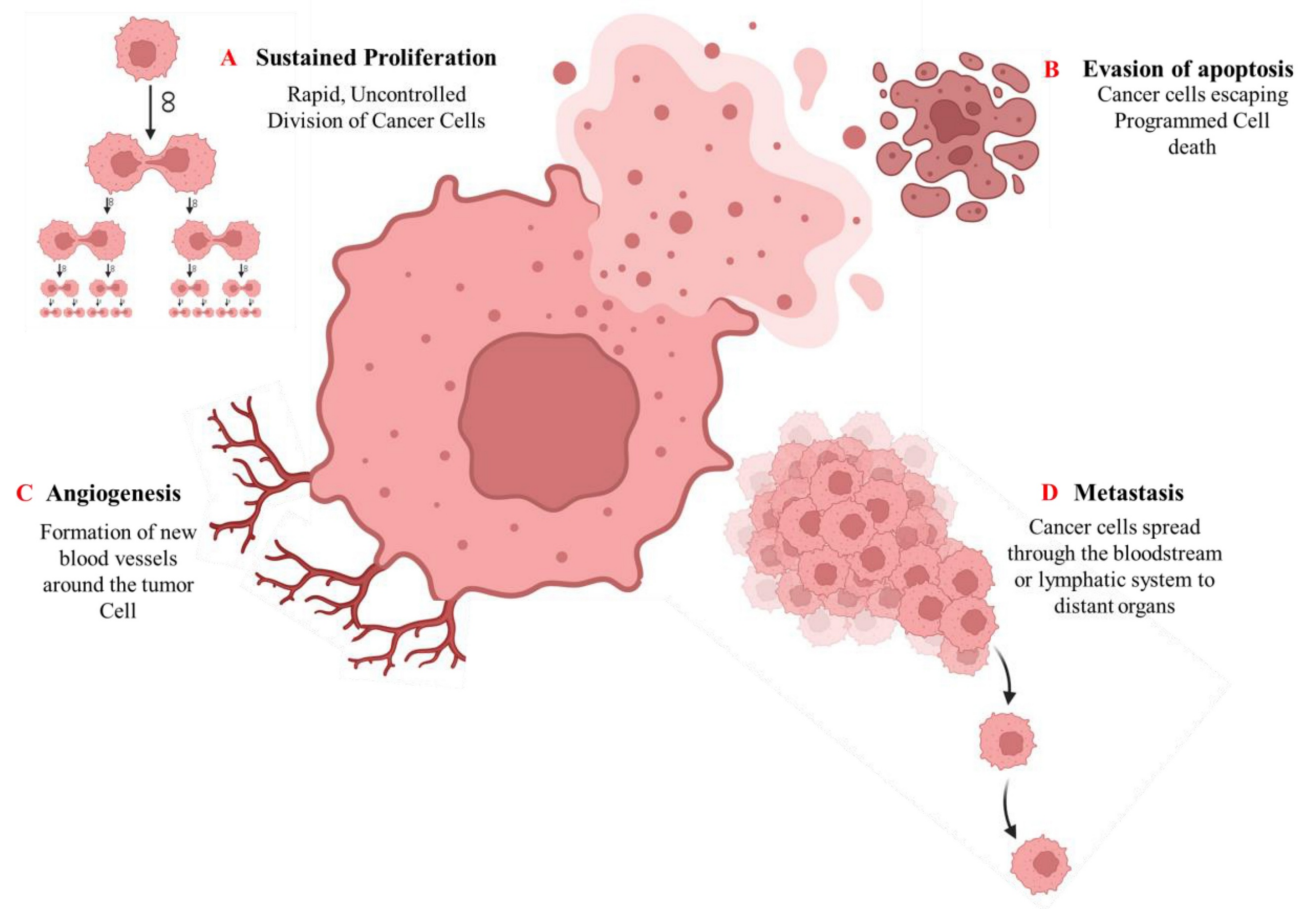
Key hallmarks of cancer pathogenesis. The diagram illustrates fundamental cellular capabilities acquired during cancer development: (A) Sustained proliferative signaling, (B) Evasion of apoptosis, (C) Induction of angiogenesis, and (D) Tissue invasion and metastasis.

Cancer pathogenesis is a highly intricate and multistep process that begins with genetic alterations and advances through the distinct but overlapping phases of tumor initiation, promotion, and progression 42. The initiation phase is characterized by the acquisition of irreversible genetic mutations within a normal cell, frequently caused by exposure to carcinogenic agents such as tobacco smoke, radiation, environmental toxins, or viral infections 43. These mutations frequently affect key regulatory genes, including tumor suppressors (such as *P53* and *RB1*) and proto-oncogenes (such as *RAS* and *MYC*), disrupting the balance between cell proliferation and programmed cell death 44. Epigenetic modifications can alter gene expression without altering the DNA sequence itself. These epigenetic processes, which include DNA methylation, histone alterations, and tiny non-coding microRNAs (miRNAs), are both heritable and reversible. Epigenetic disruptions can affect gene function and lead to malignant cell transformation 45. During the promotion phase, initiated cells undergo selective clonal expansion driven by both intrinsic genetic instability and extrinsic microenvironmental factors. Key promoters include hormones, growth factors, chronic inflammation, and tumor-promoting agents that facilitate uncontrolled proliferation 46. The progression phase involves the transition from benign lesions to invasive, metastatic malignancies. At this stage, cancer cells acquire a series of functional capabilities, often referred to as cancer hallmarks, that involve persistent proliferative signaling, evasion of growth suppression, resistance to apoptosis, replicative immortality, angiogenesis induction, tissue invasion, and metastasis 42, 47, as shown in (Fig. 3).

## 2. Different Pathways Involved in Cancer

Cancer progression is affected by molecular pathways that control cellular growth, division, and survival. Cancer cells can bypass normal regulatory controls when these pathways become dysregulated 32. Dysregulation often arises from mutations in key regulatory genes, epigenetic alterations, or the activation of aberrant signaling pathways 48. The PI3K/AKT/mTOR pathway plays a role in other essential cellular functions, including blood vessel formation, growth, and metabolism 49. Meanwhile, the Ras/Raf/MEK/ERK cascade encourages ongoing cell growth and resistance to signals that inhibit proliferation 50. Similarly, the JAK-STAT signaling pathway is a crucial cell-survival mechanism that regulates gene expression in inflammation, immunology, and cancer. The major components of this signaling cascade include cytokine receptors, signal transducer and activator of transcription (STAT) proteins, and Janus kinases (JAK) 51. These molecular abnormalities not only promote primary tumor growth but also facilitate angiogenesis, invasion, and metastasis, ultimately resulting in therapeutic resistance and poor clinical outcomes 52.

### 2.1 PI3K/Akt/mTOR

**Figure 4:**
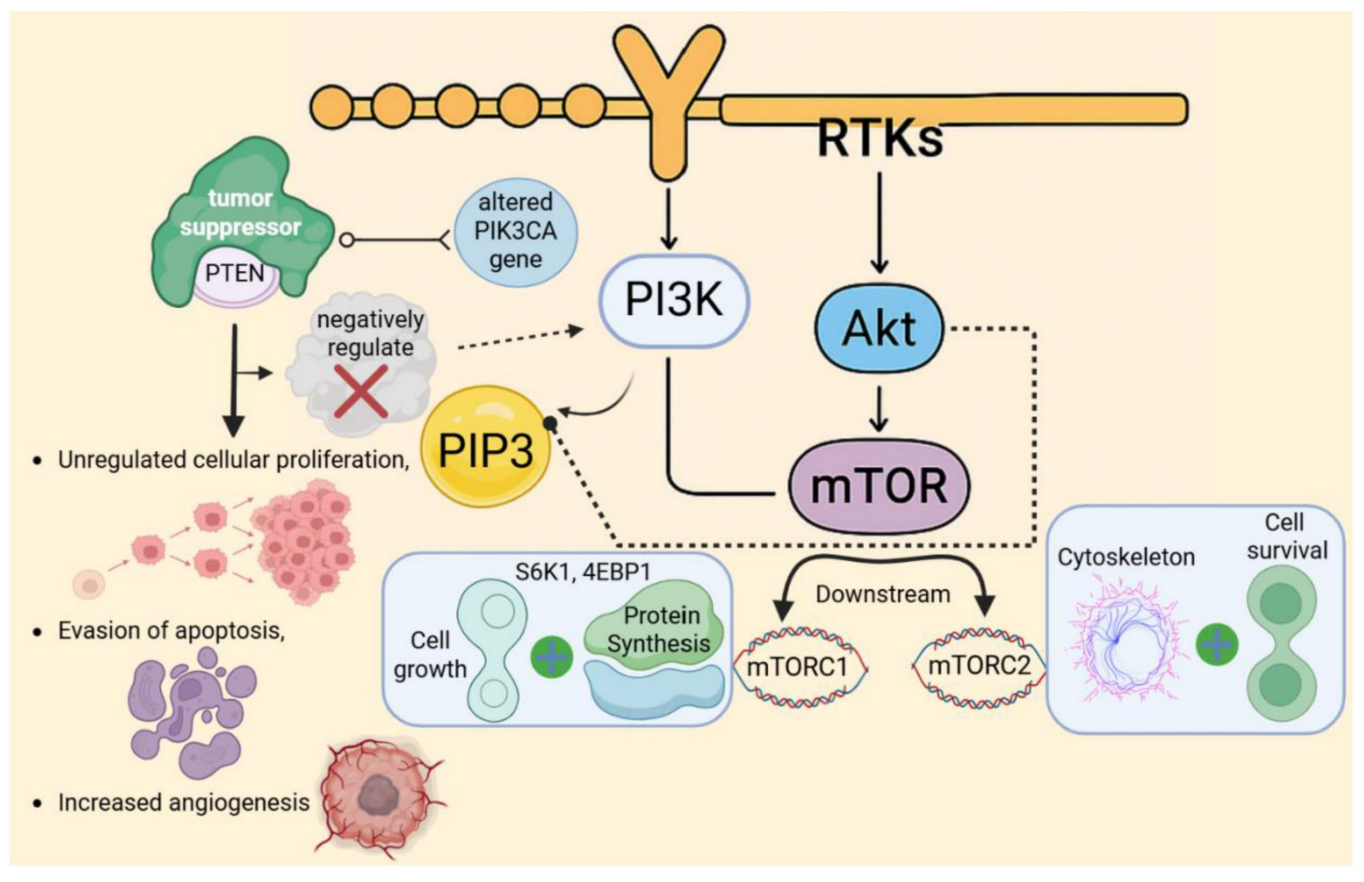
Hyperactivation of the PI3K/Akt/mTOR pathway drives oncogenic signaling. In cancer, mutations in PIK3CA or loss of PTEN lead to constitutive pathway activation, promoting unchecked cell survival, proliferation, and metabolic reprogramming. This dysregulation is a frequent therapeutic target in numerous malignancies.

Phosphoinositide 3-Kinase (PI3K) is a lipid kinase that is primarily activated by the binding of growth factors with receptor tyrosine kinases (RTKs) located on the cell membrane, including receptors, for instance, the EGFR (epidermal growth factor receptor) and IGF-1R (insulin-like growth factor receptor) 53. Upon activation, PI3K catalyzes the production of phosphatidylinositol-3,4,5-triphosphate (PIP3), a lipid second messenger that is crucial for recruiting various proteins to the cell membrane 54. PIP3 recruits many important proteins, including Akt (Protein Kinase B), which is activated at the membrane by phosphorylation 55. Once active, Akt promotes cellular survival and proliferation by phosphorylating and deactivating many pro-apoptotic proteins, including Bad and FOXO 56. Furthermore, Akt operates on mTOR (Mammalian Target of Rapamycin), a downstream effector that is divided into two complexes: mTORC1 and mTORC2 57. Akt activates mTORC1 to enhance protein synthesis and cellular growth by phosphorylating S6K1 and 4EBP1, both of which are required for mRNA translation. In contrast, mTORC2 modulates the cytoskeleton and promotes cell survival 58. The PI3K/Akt/mTOR signaling system plays a significant role in cancer formation, primarily through mutations or hyperactivation of its components 53. Additionally, shifts in the phosphatidylinositol-4,5-bisphosphate 3-kinase catalytic subunit alpha (PIK3CA) gene, which encodes the catalytic subunit of PI3K, along with shifts in PTEN, a tumor suppressor that negatively regulates PI3K activity, and Akt, have been identified in multiple cancers 58. These alterations result in unregulated cellular proliferation, evasion of apoptosis, and increased angiogenesis, all of which facilitate tumor growth and persistence. Given its pivotal function in cancer biology, the PI3K/Akt/mTOR has emerged as a critical target for therapeutic intervention 57. Current and emerging pharmacological agents, including PI3K inhibitors such as alpelisib, Akt inhibitors, and mTOR inhibitors like everolimus and rapamycin, are being used or developed to disrupt this pathway and effectively impede cancer progression 32 (Fig. 4).

### 2.2 Wnt/β-catenin

**Figure 5:**
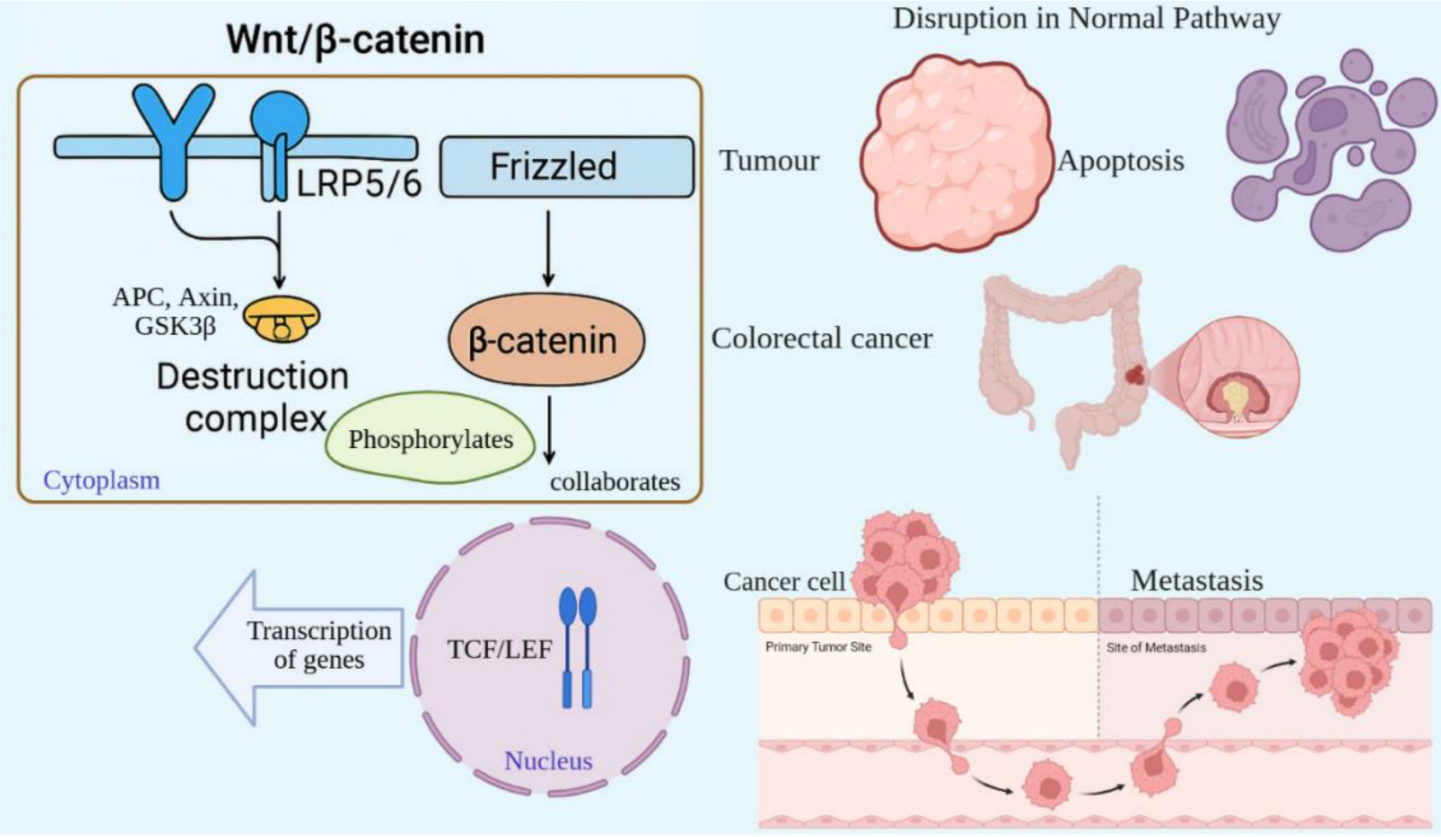
Dysregulated Wnt/β-catenin signaling promotes tumorigenesis and stemness. In the absence of Wnt, β-catenin is degraded (left). Wnt activation stabilizes β-catenin, enabling nuclear translocation and transcription of pro-growth genes (right). In cancers such as colorectal carcinoma, mutations in APC or CTNNB1 cause constitutive activation, fueling proliferation, metastasis, and cancer stem cell maintenance.

The Wnt/β-catenin pathway is a highly conserved signaling pathway that controls embryonic development, maintains tissue homeostasis, and promotes cell proliferation. Abnormalities in this pathway are linked with cancers and other diseases 59. Wnt proteins activate the pathway, which is a group of secreted signaling molecules that bind to Frizzled (FZD) receptors and their co-receptors, LRP5/6, located on the cell membrane 60. In the absence of Wnt signaling, β-catenin is degraded by a "destruction complex" consisting of APC, Axin, and GSK3β 61. This complex phosphorylates β-catenin, leading to its proteasomal degradation. Conversely, when Wnt ligands bind their receptors, the destruction complex is inhibited, halting the degradation of β-catenin 62. As a result, β-catenin aggregates in the cytoplasm and translocates to the nucleus, where it collaborates with transcription factors such as TCF/LEF to activate the transcription of genes targeted by Wnt signaling 63. The Wnt/β-catenin signaling pathway is tightly regulated in healthy cells; however, its disruption can lead to unchecked cellular proliferation and tumor formation 60. Frequently, mutations in key components of this pathway, such as APC or β-catenin, are observed in colorectal cancer 64. These mutations inhibit the degradation of β-catenin, leading to its persistent activation and promoting the expression of genes associated with cell cycle progression and survival 59. In cancer, improper stimulation of Wnt/β-catenin signaling promotes tumor development, resistance to cell death, and metastasis. Furthermore, this pathway plays a role in maintaining cancer stem cells, which are involved in tumor recurrence 63 (Fig. 5).

### 2.3 MAPK/ERK

**Figure 6:**
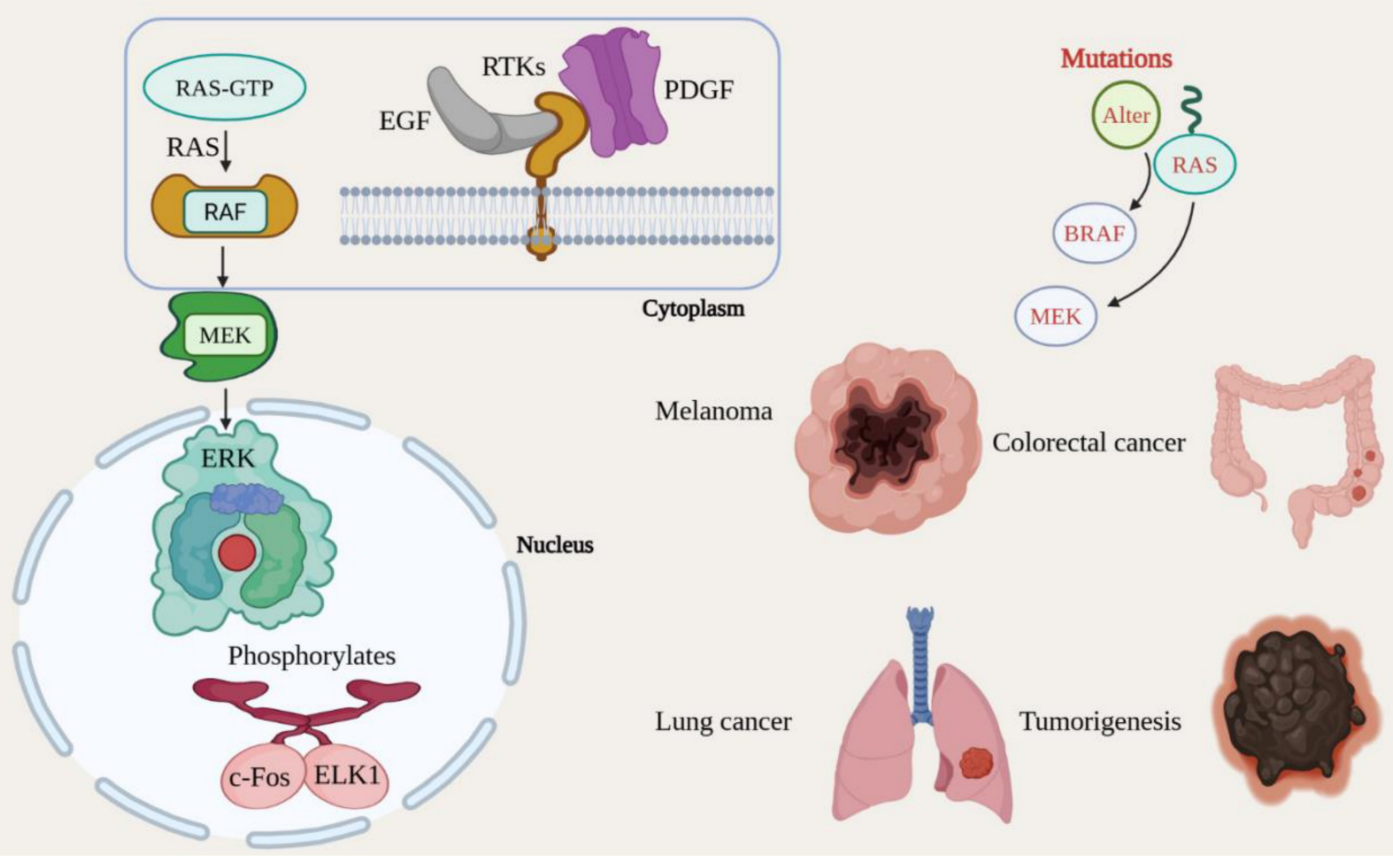
Oncogenic mutations constitutively activate the MAPK/ERK proliferation cascade. Growth factor binding initiates a kinase cascade (RAS→RAF→MEK→ERK) that regulates cell cycle progression. Gain-of-function mutations in RAS, BRAF (e.g., V600E), or MEK result in ligand-independent signaling, driving uncontrolled growth in melanoma, lung, and colorectal cancers.

The Mitogen-Activated Protein Kinase/Extracellular Signal-Regulated Kinase (MAPK/ERK) signaling pathway is a crucial mechanism that governs a range of cellular functions, including proliferation, differentiation, survival, and development 65. Growth factors and other mitogenic signals predominantly activate this pathway, and its dysregulation is commonly linked with cancer 66. The MAPK/ERK pathway is initiated when growth factors, including Epidermal Growth Factor (EGF) and Platelet-Derived Growth Factor (PDGF), bind to RTKs (receptor tyrosine kinases) located on the cell membrane, including the EGFR 67. The binding of these growth factors leads to the activation of the small GTPase RAS, which transitions from its inactive GDP-bound state to its active GTP-bound state. As a result, active RAS recruits and activates RAF, a serine/threonine kinase, which subsequently phosphorylates and promotes MEK (MAPK/ERK kinase) 68. MEK, in turn, phosphorylates and activates the final kinase in the signaling cascade, ERK (Extracellular signal-regulated Kinase). Once activated, ERK translocates to the nucleus and phosphorylates numerous transcription factors, including c-Fos and ELK1, thereby increasing the transcription of genes essential for cell cycle progression, proliferation, and survival 32. The MAPK/ERK signaling system is frequently mutated in malignancies, leading to chronic activation and uncontrolled cellular proliferation 69. Changes in critical components of this system, including RAS, BRAF, and MEK, are seen in a variety of cancer forms, including melanoma, colorectal cancer, and lung cancer. Notably, the BRAF V600E mutation is associated with sustained ERK activation, which facilitates tumorigenesis 70. The aberrant regulation of the MAPK/ERK pathway fosters cancer cell growth, survival, invasion, and metastasis. Additionally, it plays a part in evasion of apoptosis, thereby further promoting tumor advancement 71 (Fig. 6).

### 2.4 p53

**Figure 7:**
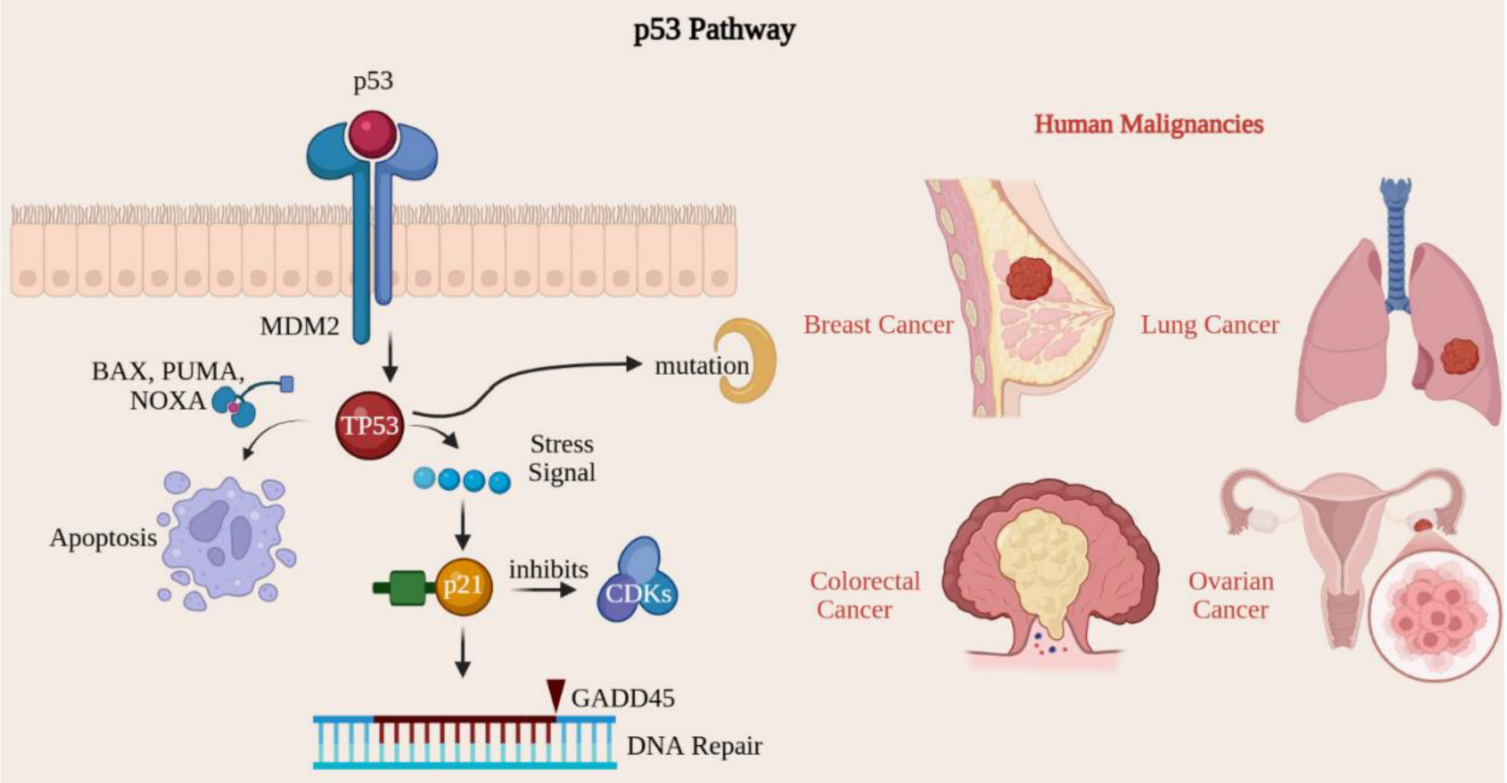
Inactivation of the p53 pathway leads to genomic instability and tumor progression. The p53 tumor suppressor coordinates cellular stress responses, inducing cell-cycle arrest or apoptosis to maintain genomic integrity. Mutations in TP53, which are common in over 50% of human cancers, disable these protective functions, allowing the accumulation of DNA damage and unchecked proliferation.

The p53 signaling pathway acts as a critical tumor suppressor, maintaining genomic integrity by regulating cell cycle, DNA repair, apoptosis, and cellular senescence 44. The TP53 gene encodes the p53 protein, known as the guardian of the genome due to its essential role in preventing cancer development 72. Mutations within the TP53 gene rank among the most prevalent genetic alterations observed in human malignancies 73. The p53 protein is activated in response to many cellular stresses, including DNA damage, oxidative stress, hypoxia, and oncogene activation. In a healthy cellular environment, p53 levels are maintained at low levels through its association with MDM2, a protein that promotes p53 degradation 74. Upon activation, p53 enhances gene expression, effectively pausing the cell cycle at the G1/S checkpoint and providing cells with time to repair DNA damage before replication 75. Notable p53 target genes in this context include p21, which inhibits cyclin-dependent kinase (CDK) activity. Additionally, p53 activates genes crucial for DNA repair, such as GADD45, thereby ensuring genomic stability by rectifying damaged DNA before cellular division occurs 44. When DNA damage is excessively severe and irreparable, p53 can trigger apoptosis by activating pro-apoptotic genes such as BAX, PUMA, and NOXA, which facilitate cell death and prevent the proliferation of potentially cancerous cells 76. p53 acts as a critical guardian of the genome, orchestrating the response to DNA damage by arresting the cell cycle for repair or initiating apoptosis to eliminate potentially oncogenic cells 76. When the TP53 gene is mutated or inactivated, cells may bypass normal growth regulatory mechanisms, leading to continued division despite DNA damage, which ultimately contributes to tumorigenesis 74, 76. Mutations in the TP53 gene have been found in more than 50% of human cancers, including lung, colorectal, breast, and ovarian 75. These alterations frequently result in the synthesis of a dysfunctional p53 protein that is unable to trigger cell cycle arrest or apoptosis, thereby permitting the unchecked proliferation of cancerous cells 77 (Fig. 7).

## 3. Metastasis

**Figure 8:**
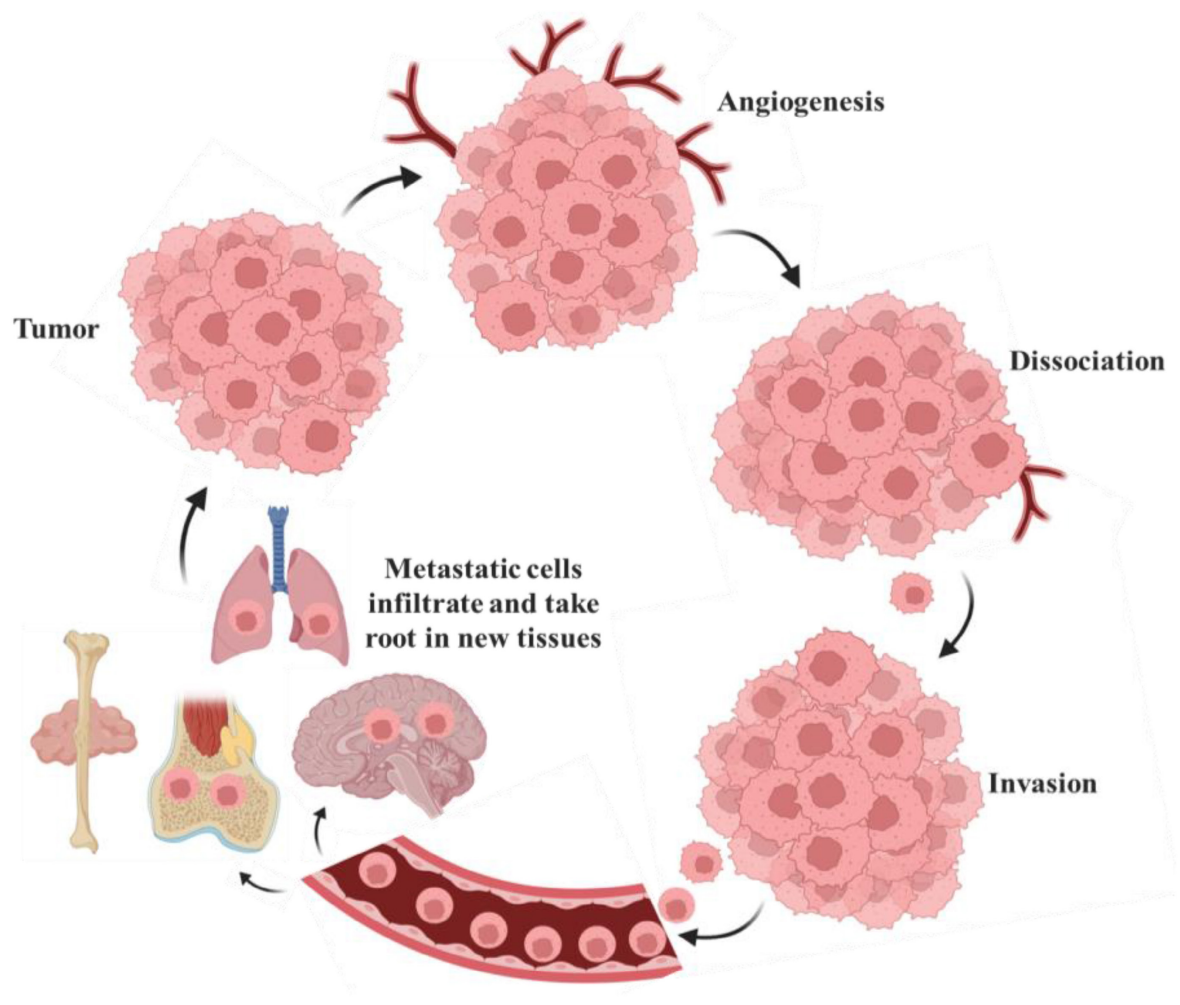
Metastasis involves cells traveling from the primary tumor to distant organs. Large tumors that experience tissue hypoxia rely on angiogenesis to facilitate the exchange of nutrients and waste through blood vessels. Cells from the primary tumor undergo phenotypic changes, including decreased cell-cell adhesion, which allows them to detach from the main mass, invade the surrounding extracellular matrix (ECM), and enter the bloodstream or lymphatic system. Once in circulation, tumor cells can extravasate and form secondary tumors, where the metastatic process can repeat itself 83.

Metastasis, the spread of cancer cells from a primary tumor to distant organs, is the leading cause of cancer-related death, as illustrated in Fig. 8. This process involves cancer cells infiltrating adjacent tissues by breaking down the extracellular matrix 78. Additionally, these cells can enter the bloodstream or lymphatic vessels, navigate the circulatory system, and evade immune detection. Once in circulation, cancer cells can leave the bloodstream, infiltrate new tissues, and form additional tumors in distant sites 79. This inclination is influenced by multiple factors, including the compatibility of cancer cells ('the seed') with the microenvironment of the target tissue ('the soil'), as articulated in the seed-and-soil hypothesis 80. This inclination is influenced by multiple factors, including the compatibility of cancer cells with the microenvironment of the target tissue, as articulated in the seed and soil hypothesis. Breast cancer spreads to the bones, liver, lungs, and brain 39. Prostate cancer shows a strong tendency to metastasize to the bones. Colon cancer often spreads to the liver, primarily due to the direct blood flow from the intestines through the portal vein 81. Lung cancer has the potential to metastasize to the brain, bones, liver, and adrenal glands 82.

## 4. Cancer Diagnosis and Treatment

As shown in Table 2, the diagnosis involves taking a full medical history, conducting a physical examination, and performing a series of tests and procedures to detect and confirm the presence of cancer, identify the type, stage, and extent of the disease, and guide treatment choices. Early and accurate diagnosis is essential for improving treatment outcomes and patient survival 84.

Cancer management involves a variety of strategies aimed at eliminating or controlling malignant cells, with treatment choices influenced by factors such as the specific type of cancer, its stage, location, and the patient's overall health 92. The main goals of treatment include curative approaches, which seek complete remission; adjuvant strategies, designed to prevent recurrence after initial therapy; and palliative care, which focuses on relieving symptoms and improving the quality of life for patients with severe or terminal illnesses. Treatment options are generally categorized into traditional methods, like surgery, chemotherapy, and radiation, and modern techniques, including targeted therapy, immunotherapy, and precision medicine. Each option has unique mechanisms of action and clinical benefits, but also presents limitations and challenges, such as systemic toxicity, the development of therapeutic resistance, and off-target effects 93. Understanding these trade-offs is essential for developing effective, personalized treatment plans. The key features, uses, and limitations of current cancer therapies are briefly summarized in Table 3.

## 5. Role of Nanoparticles in Cancer Treatment and Diagnosis

Nanoparticles are engineered to serve as highly sensitive diagnostic probes that can detect cancer at its earliest stages, thereby providing significant benefits in disease management 108. These minuscule particles enhance the targeted delivery of therapeutic agents directly to cancerous cells, mitigating treatment resistance and improving therapy efficacy, as shown in Table 4.

### 5.1 Mechanistic overview of nanoparticle action in cancer

The therapeutic and diagnostic efficacy of nanoparticles in oncology is underpinned by several interrelated biological and physicochemical mechanisms, which can be broadly categorized into passive and active targeting strategies. Initially, systemically administered nanoparticles preferentially accumulate in tumor tissue through the enhanced permeability and retention (EPR) effect, a passive targeting phenomenon driven by the leaky, disorganized vasculature and impaired lymphatic drainage characteristic of solid tumors 117, 118. This provides a foundational, albeit variable, degree of tumor selectivity based on size and circulation time. To overcome the limitations of passive targeting and enhance specificity, NPs can be actively targeted by surface functionalization with ligands (e.g., antibodies, peptides, aptamers) that recognize and bind receptors overexpressed on cancer cells or on the associated vasculature. This ligand-receptor interaction not only increases tumor accumulation but also facilitates receptor-mediated endocytosis, promoting cellular internalization of the NP payload 119, 120. The choice between passive and active targeting or their combination depends on the tumor type, vascularization, and the specific therapeutic or diagnostic objective.

Once internalized, NPs must navigate intracellular pathways. Many are entrapped within endo-lysosomal compartments, where the acidic pH or specific enzymes can trigger stimulus-responsive drug release. Advanced NPs are engineered with "smart" materials that release their therapeutic cargo in response to unique tumor microenvironment cues, such as low pH, elevated glutathione (redox potential), or overexpressed enzymes (e.g., matrix metalloproteinases) 121, 122. Beyond drug delivery, certain NPs exert direct anticancer effects. For example, inorganic nanoparticles (e.g., gold, iron oxide) can convert near-infrared light into heat for photothermal ablation or generate reactive oxygen species in photodynamic therapy 123, 124. Furthermore, NPs can be designed to reverse multidrug resistance by co-delivering chemotherapeutic agents with efflux pump inhibitors or by bypassing efflux mechanisms altogether 125. Collectively, these mechanisms, passive and active targeting, controlled internalization, stimuli-responsive release, and direct physical action, enable nanoparticles to improve therapeutic indices, minimize off-target effects, and overcome biological barriers that limit conventional therapies 126, 127.

### 5.2 Clinical translation and challenges

By specifically targeting malignant cells, nanoparticles minimize adverse effects on healthy tissues and improve the precision of both therapeutic and diagnostic approaches 128. This advancement not only increases treatment accuracy but also facilitates earlier cancer detection, which is vital for improving patient outcomes and advancing cancer treatment strategies 129. The clinical trials and approved agents outlined in Table 5 highlight several significant trends in oncological nanomedicine. Initially, early achievements were primarily characterized by passively targeted platforms, such as liposomes (e.g., Doxil®), which exploit the enhanced permeability and retention (EPR) effect, with the main goal of improving the therapeutic index of existing chemotherapeutics 130. However, the current pipeline is swiftly transitioning towards actively targeted and multifunctional systems. These advanced nanoparticles are engineered for specific receptor-mediated uptake (e.g., MM-310), the co-delivery of synergistic agents, or immunostimulants (e.g., mRNA cancer vaccines) 131. Additionally, the emergence of lipid nanoparticles (LNPs), as demonstrated by their effectiveness in mRNA COVID-19 vaccines, has opened powerful new avenues for cancer immunotherapy and gene editing. This technological validation confirms the utility of LNPs for the efficient and safe delivery of diverse therapeutic payloads, including cancer-specific antigens, immunomodulators, and gene-editing components, paving the way for personalized cancer vaccines and targeted immunotherapies 132-134. Nevertheless, despite these advancements, the clinical translation of nanotherapeutics encounters obstacles, including issues related to batch-to-batch reproducibility, scalability, and the need to demonstrate clear advantages over standard-of-care treatments in randomized trials. The future of this domain is centered on the development of genuinely intelligent, theranostic platforms capable of diagnosing, treating, and monitoring responses in real-time 135.

## 6. Prevention Strategies

Cancer prevention strategies aim to reduce disease incidence by encouraging lifestyle changes, such as quitting smoking, improving dietary habits, and engaging in regular physical activity 139. Prophylactic medical interventions, including immunizations against oncogenic viruses, offer crucial protection; for instance, the HPV vaccine reduces the incidence of cervical, anal, and oropharyngeal cancers 140, while the hepatitis B vaccine decreases the occurrence of hepatocellular carcinoma 141. In addition to these programs, standard population screenings, such as mammograms for breast cancer, Pap smears for cervical cancer, and colonoscopies for colorectal cancer, help in early diagnosis and improve treatment results 142. Collectively, these primary, secondary, and tertiary prevention measures form the cornerstone of reducing the global cancer burden.

## 7. Future Perspectives

Beyond current modalities, the oncology landscape is poised for transformation through several converging technological advances. Precision medicine, powered by whole-genome sequencing, enables the discovery of driver mutations and helps develop personalized therapies that optimize efficacy and reduce toxicity 143. Gene-editing technologies, particularly CRISPR-Cas9, offer remarkable opportunities to directly correct harmful genetic changes 144 and are being explored in clinical trials for cancers such as leukemia and melanoma 26. Nanotechnology continues to advance targeted drug delivery, enhancing therapeutic indices by increasing local drug concentrations while minimizing systemic side effects 145. Furthermore, nanosensors are being developed for highly sensitive detection of circulating biomarkers, enabling earlier diagnosis 14.

A central challenge for next-generation nanomedicine is targeting tumor heterogeneity and the complex immunosuppressive tumor microenvironment (TME). A significant focus in oncology is the tumor microenvironment (TME), which comprises non-cancerous cells such as cancer-associated fibroblasts (CAFs), immune cells, blood vessels, and signaling molecules 146. The TME plays a dual role, initially suppressing tumor growth but often being co-opted by cancer cells to promote proliferation, invasion, metastasis, and therapy resistance. The immunosuppressive nature of the TME, characterized by regulatory T cells (Tregs) and M2-type tumor-associated macrophages (TAMs), is a major barrier to immunotherapy. Therefore, next-generation therapies, including nanomedicines, are being designed not only to target cancer cells but also to reprogram the TME, for instance, by re-educating TAMs toward an anti-tumor M1 phenotype or by disrupting the supportive stromal network 147, 148. Future nano-platforms must be designed to penetrate dense stromal matrices and reach hypoxic tumor cores. Strategies include reprogramming the TME by modulating cancer-associated fibroblasts (CAFs) or shifting TAMs (tumor-associated macrophages) from a pro-tumor (M2) to an anti-tumor (M1) phenotype. The ultimate objective is to develop intelligent, multifunctional theranostic agents that integrate real-time diagnostic imaging (e.g., MRI, fluorescence) with stimulus-triggered drug release. These systems would enable clinicians to monitor drug delivery, evaluate treatment responses, and adapt strategies in real time, paving the way for a truly personalized and responsive approach to cancer treatment.

## 8. Conclusion

Cancer is a complex array of diseases shaped by both genetic and environmental factors. While traditional treatment options, such as surgery, chemotherapy, and radiation, have resulted in enhanced survival rates for patients, they frequently face limitations due to toxicity and the emergence of drug resistance. The introduction of nanoparticles marks a significant advancement, enabling targeted drug delivery, enhanced imaging, and innovative therapeutic strategies, such as photothermal and photodynamic therapies, that aim to minimize harm to healthy tissues. However, challenges remain, including the toxicity of nanoparticles and the difficulties associated with their clinical implementation. The integration of cutting-edge molecular biology, nanotechnology, and artificial intelligence is ushering in a new era in cancer treatment. Shifting away from conventional, non-specific therapies, the future of oncology is focused on personalized, integrated strategies that emphasize early detection, targeted delivery, and resolving therapeutic resistance. The integration of nanotechnology with AI-driven diagnostics and personalized medicine holds promise for transforming cancer into a manageable chronic disease, ultimately improving survival and quality of life for patients worldwide.

## Figures and Tables

**Table 1:** Common Types of Cancer and Their Characteristics

Cancer Type	Description	Examples	Common Risk Factors	Common Metastatic Sites	References
Carcinomas	The most frequent kind of cancer is caused by epithelial cells, which line the body's interior and exterior surfaces, and account for 80-90% of all cancer cases.	Adenocarcinoma (breast, colon, pancreas, prostate, lungs). Squamous Cell (skin, cervix, head, and neck). Transitional Cell (bladder). Basal Cell (skin). Renal Cell (kidney)	Smoking (lung). UV exposure (skin). High red meat consumption (colorectal cancer). Genetic mutations (BRCA1/BRCA2 for breast and ovarian cancer). HPV infection (cervical carcinoma).	Lungs, liver, bones, and brain.	8, 13
Sarcomas	A rare type of cancer arising from mesenchymal cells affects connective tissues, including bone, muscle, cartilage, and fat. Represents about 1% of cancers in adults.	Liposarcoma (fat). Leiomyosarcoma (smooth muscle). Rhabdomyosarcoma (skeletal muscle). Osteosarcoma (bone). Ewing sarcoma (bone)	Genetic mutations (e.g., Li-Fraumeni syndrome). Radiation exposure.	Lungs.	15, 16
Leukemias	Cancers that damage the blood-forming tissues (bone marrow and lymphatic system), resulting in an excess of aberrant blood cells, particularly white blood cells.	Acute Lymphoblastic (ALL). Acute Myeloid (AML). Chronic Lymphocytic (CLL). Chronic Myeloid (CML)	Genetic mutations. Exposure to chemicals like benzene. Radiation exposure.	Central Nervous System (for some types).	20, 30
Lymphomas	Cancers develop in the lymphatic system, namely in lymphocytes (a kind of white blood cell). Hodgkin's and non-Hodgkin's lymphomas are the two main types.	Hodgkin Lymphoma (HL): Includes Classical Hodgkin Lymphoma (cHL) and Nodular Lymphocyte-Predominant Hodgkin Lymphoma (NLPHL). Non-Hodgkin Lymphoma (NHL): Includes Diffuse Large B-cell lymphoma (DLBCL), Follicular Lymphoma, and Burkitt Lymphoma.	Weakened immune system (HIV/AIDS). Infections like Epstein-Barr virus (EBV). Immunosuppressive drugs.	Liver, spleen, bone marrow	22, 31
Melanomas	The most lethal type of skin cancer originates in melanocytes (cells that produce melanin). Though rare, it has a high metastatic potential if not detected early.	Superficial Spreading (most common). Nodular (aggressive). Lentigo Maligna (slow-growing, in sun-exposed areas). Acral Lentiginous (rare, found on palms and soles).	UV radiation exposure. Fair skin, light hair, and freckling. Family history of melanoma.	Lungs, liver, brain, and bones.	26, 28

**Table 2:** List of Diagnostic methods used for the diagnosis of Cancer

Diagnostic Method	Description	Examples/Tools	References
Medical History and Physical Examination	The initial step is to assess symptoms, medical history, family history, and lifestyle factors. Physical examination checks for abnormal lumps or signs.	Lumps in breast cancer, enlarged lymph nodes in lymphoma.	39, 85
Imaging Techniques	Used to view tumors and determine their size, location, and spread.	X-rays: Detect bone/lung cancers. Ultrasound: Detects soft tissue masses. CT scan: Detailed cross-sectional images. MRI: For soft tissue tumors like the brain/spinal cord. PET Scan: Detects metabolic activity of tumors.	86, 87
Laboratory Tests	Blood and urine tests detect abnormal markers or signs of organ dysfunction.	Tumor markers: PSA (prostate), CA-125 (ovarian), AFP (liver). Blood cell counts abnormalities (leukemia, lymphoma).	88
Biopsy	Removal and examination of tissue samples to confirm the presence of cancer.	Needle Biopsy: Extracts a small tissue sample, guided by imaging. Surgical Biopsy: Partial or complete tumor removal. Endoscopic Biopsy: Performed during procedures like colonoscopy or bronchoscopy.	89
Molecular and Genetic Testing	Detects genetic mutations and chromosomal changes in cancer cells.	FISH/PCR: Detect HER2 (breast cancer) and BCR-ABL (CML). Next-Generation Sequencing (NGS): Identifies mutations for targeted therapy.	90
Staging and Grading	Determines the extent and aggressiveness of cancer.	TNM Staging evaluates tumor size (T), lymphatic involvement (N), and metastasis (M). Grading: Looks at how abnormal cancer cells appear.	91

**Table 3:** Structured view of various cancer treatment methods, their descriptions, and common uses

Treatment Method	Description	Uses	Limitations	References
Surgery	Surgical excision of the tumor, along with adjacent tissues, when necessary, is the Primary approach for treating localized cancers.	Breast cancer treatment options include lumpectomy and mastectomy. Colectomy for colorectal cancer. Prostatectomy is used to treat prostate cancer.	Not effective for metastatic or disseminated disease. Risk of postoperative complications (infection, bleeding). Potential for disfigurement or loss of organ function. Risk of incomplete resection if margins are positive.	94
Radiation Therapy	Utilizes high-energy radiation to eradicate or impair cancerous cells and reduce tumor size. This treatment may be administered before, following, or as an alternative to surgical intervention.	External Beam Radiation: Focuses radiation on specific areas, e.g., breast, brain, and prostate cancers. Internal Radiation (Brachytherapy): Inserts radioactive material into the body; typically used for prostate and cervical malignancies.	Off-target damage to surrounding healthy tissues, causing acute and long-term side effects (e.g., fibrosis, secondary malignancies). Tumors can develop radio resistance.- Limited efficacy in hypoxic tumor regions.	95, 96
Chemotherapy	A systemic therapy that employs medications to destroy quickly dividing cancer cells. It can be used alone or in conjunction with other treatments.	Cisplatin and Carboplatin for lung and ovarian cancers. Cyclophosphamide for leukemia and lymphoma. Doxorubicin for breast cancer.	Systemic toxicity to healthy proliferating cells (e.g., bone marrow suppression, gastrointestinal distress, alopecia). Development of multidrug resistance (MDR). Narrow therapeutic index.	97, 98
Targeted Therapy	Drugs are created to target particular chemicals involved in cancer cell proliferation and survival. In comparison to chemotherapy, it is often less damaging to normal cells.	Herceptin (trastuzumab) for HER2-positive breast cancer. Erlotinib is used to treat lung cancer with an EGFR mutation. Gleevec (Imatinib) for chronic myeloid leukemia (CML).	Tumors can develop resistance through secondary mutations or alternate signaling pathways. High cost and accessibility issues. Off-target effects can still occur (e.g., skin rash, cardiac toxicity).	99
Immunotherapy	Increases or changes the immune system's ability to identify and kill cancer cells.	Checkpoint inhibitors include pembrolizumab (Keytruda) and nivolumab for melanoma, lung cancer, and bladder cancer. CAR-T Cell Therapy: Used for leukemia and lymphoma.	Can induce immune-related adverse events (irAEs) (e.g., colitis, pneumonitis, endocrine dysregulation). Only a subset of patients responds (biomarker needed). High cost and complex manufacturing (e.g., for CAR-T) (Risk of cytokine release syndrome (CRS) and neurotoxicity).	100
Hormone Therapy	Blocks or lowers the number of hormones (e.g., estrogen or testosterone) that fuel certain cancers' growth. Often used in hormone-sensitive cancers.	Tamoxifen for estrogen receptor-positive breast cancer. Leuprolide (Lupron) for prostate cancer. Aromatase inhibitors (Anastrozole, Letrozole) for postmenopausal women with breast cancer.	Tumors can evolve hormone-independent growth mechanisms. Side effects include menopause-like symptoms, osteoporosis, and increased risk of thromboembolism.	101
Bone Marrow Transplant (Stem Cell Transplant)	Replaces damaged or destroyed bone marrow with healthy stem cells, usually after high-dose chemotherapy or radiation.	Used in leukemia, lymphoma, and multiple myeloma. Autologous (patient's cells) or Allogeneic (donor cells) transplants.	Graft-versus-host disease (GVHD) occurs after allogeneic transplants. High risk of life-threatening infections due to prolonged immunosuppression. Transplant-related mortality.	102
Photodynamic Therapy (PDT)	Kills cancer cells by combining photosensitizing chemicals with light. Effective for treating cancers close to the skin or internal linings.	Used for skin, esophageal, and lung cancer (non-small cell) in early stages.	Limited to superficial or endoscopically accessible tumors due to limited light penetration. Causes photosensitivity in patients post-treatment.	103
Cryotherapy (Cryosurgery)	Uses extreme cold to freeze and destroy cancerous tissues. Typically used for small or localized cancers.	Effective for early-stage skin cancer, cervical cancer, and prostate cancer.	Risk of damage to adjacent healthy structures and nerves. Potential for inadequate freezing leading to local recurrence.	104
Hyperthermia Therapy	Involves exposing body tissues to high temperatures to damage and kill cancer cells while minimizing harm to normal cells. Often used with other treatments like radiation.	Used for sarcoma, breast cancer, and cervical cancer. Helps enhance the effectiveness of radiation therapy.	Technically challenging to achieve and monitor uniform heating of the tumor. Risk of burns to surrounding healthy tissues.	105
Precision Medicine	A personalized treatment approach that uses genetic and molecular analysis to tailor therapies to the individual patient's cancer profile.	Next-Generation Sequencing (NGS) identifies mutations for targeted therapy in various cancers, like lung and colorectal cancer.	Tumor heterogeneity can lead to treatment resistance. Access to sequencing and the high cost of targeted drugs. Finding actionable mutations is not always possible.	106
Palliative Care	Patients with advanced or incurable malignancies benefit from treatment that alleviates symptoms and improves their quality of life.	Used in advanced cancers for symptom management, such as pain control, fatigue, nausea, and emotional support.	Often underutilized and referred to too late in the disease course. Not a curative treatment, but a supportive measure.	107

**Table 4:** Applications and Challenges of Nanoparticles in Cancer Treatment

Application	Description	Examples	References
Nanoparticles in Drug Delivery	Nanoparticles can deliver drugs directly to tumor cells, limiting injury to healthy organs while improving chemotherapeutic efficiency.	Liposomes: i.e., Doxil (liposomal doxorubicin) for targeted chemotherapy. Polymeric nanoparticles: Controlled drug release. Gold nanoparticles: Release drugs in response to light/heat. Magnetic nanoparticles: Directed by external magnetic fields.	109
Targeted Therapy Using Nanoparticles	Functionalized nanoparticles can detect and attach selectively to cancer cells, delivering deadly medications directly to tumor cells while preserving healthy tissue.	Antibody-Conjugated Nanoparticles: E.g., HER2-conjugated nanoparticles for targeting HER2-positive breast cancer cells. RNA Interference (RNAi) Therapy: Nanoparticles delivering siRNA to silence oncogenes.	39
Nanoparticles in Photothermal and Photodynamic Therapy	Nanoparticles are used in combination with light-based therapies to destroy cancer cells.	Photothermal Therapy (PTT): Gold nanoparticles convert near-infrared light into heat to kill cancer cells. Photodynamic Therapy (PDT): Nanoparticles deliver photosensitizers that generate reactive oxygen species (ROS) when activated by light.	110, 111
Nanoparticles for Overcoming Drug Resistance	Nanoparticles can bypass cancer drug resistance by delivering multiple drugs or releasing them in response to specific stimuli.	Multidrug-Resistant Cancer: Nanoparticles encapsulating both chemotherapeutic agents and efflux pump inhibitors. Smart Nanoparticles: Release drugs only in response to pH, temperature, or enzyme levels. Nanoparticles targeting cancer stem cells (CSCs) to prevent recurrence and resistance.	112
Nanoparticles in Early Cancer Detection	Nanoparticles serve as highly sensitive diagnostic tools capable of identifying cancer in its early stages, improving prognosis and survival rates.	Quantum Dots (QDs): Fluorescent nanoparticles attached to antibodies target cancer biomarkers, providing high-resolution imaging for early detection. Gold Nanoparticles (AuNPs): Used in imaging modalities like SERS and CT scans for non-invasive tumor detection.	113, 114
Challenges and Future Directions	Nanoparticles in cancer therapy face challenges related to toxicity, tumor heterogeneity, and clinical translation.	Toxicity and Biocompatibility: Ensuring safe elimination from the body. Tumor Heterogeneity: Developing multifunctional nanoparticles to target different tumor aspects. Clinical Translation: Scaling up production and meeting regulatory requirements.	115, 116

**Table 5:** Selected Clinical Trials and Approved Therapies Involving Nanoparticles in Oncology

Drug Name	Nanoparticle Platform	Target Cancer	Mechanism of Action	Phase	Status	NCT Identifier / Reference
Doxil®/Caelyx® (Liposomal Doxorubicin)	PEGylated Liposome	Ovarian, Breast, Kaposi's Sarcoma	Chemotherapeutic encapsulation; Enhanced Permeability and Retention (EPR) effect	Approved (FDA 1995)	First approved nano-drug; reduces cardiotoxicity	136
Abraxane® (Paclitaxel-albumin bound)	Albumin-bound	Breast, Pancreatic, Non-Small Cell Lung Cancer	Albumin receptor (gp60)-mediated endothelial transcytosis	Approved (FDA 2005)	Superior efficacy vs. solvent-based paclitaxel	137
Onivyde® (Irinotecan Liposome)	Liposomal	Metastatic Pancreatic Cancer	Topoisomerase inhibitor; EPR effect	Approved (FDA 2015)	Used in combination with 5-fluorouracil and leucovorin	138
mRNA-4157 (V940)	Lipid Nanoparticle (LNP)	Melanoma (adjuvant setting)	Personalized cancer vaccine encoding neoantigens	Phase III	Combined with Keytruda® (pembrolizumab); Positive Phase II results	NCT05933577
BNT122 (RO7198457)	Liposomal	Colorectal Cancer, Melanoma	Personalized mRNA-based cancer vaccine targeting patient-specific mutations	Phase II	In combination with anti-PD-L1 immunotherapy	NCT04486378
AZD0464	Dendrimer	Lymphomas, Advanced Solid Tumors	Drug conjugate delivering a Bcl-2/xL inhibitor	Phase I/II	Designed to improve the therapeutic index and reduce toxicity	NCT04214093
CRLX101	Cyclodextrin-based Polymer	Gynecologic Cancers, Renal Cell Carcinoma	Nanoparticle containing camptothecin (topoisomerase I inhibitor)	Phase II	Active targeting potential; promotes drug stability	NCT01652079
AuroLase Therapy	Gold Silica Shell (Nanoshells)	Head and Neck Cancer	Photothermal ablation; nanoshells heated with near-infrared light	Pilot Study	Localized thermal destruction of tumors	NCT00848042
MM-310	Liposomal	Solid Tumors (e.g., Ovarian, Gastric)	Liposome encapsulating a docetaxel prodrug; targets EphA2 receptor	Phase I	Active targeting to overcome limitations of passive EPR	NCT03076372
CALAA-01	Cyclodextrin-containing Polymer	Solid Tumors	RNAi nanoparticle targeting ribonucleotide reductase M2 (RRM2)	Phase I (Discontinued)	First-in-human trial of targeted siRNA delivery; demonstrated proof-of-concept	NCT00689065
